# Safety Evaluation of an Aqueous Root and Leaf Extract of Ashwagandha ( *Withania somnifera* )

**DOI:** 10.1002/ptr.70314

**Published:** 2026-04-15

**Authors:** Mukesh Summan, Candace L. Doepker, Gregory P. Brorby

**Affiliations:** ^1^ Kerry Group, Global Technology & Innovation Centre Naas Co. Kildare Ireland; ^2^ ToxStrategies Cincinnati Ohio USA; ^3^ ToxStrategies San Rafael California USA

**Keywords:** ashwagandha, botanical safety, toxicology, *Withania somnifera*

## Abstract

Consumer interest in botanicals as part of their diet continues to grow with many options being offered, but the public health requirement for an understanding of potential toxicity underscores the need for safety data to be developed specific to each form (i.e., plant part and extraction method). Regulatory agencies require standard toxicology tests to be documented in the public domain to assist with the transparency of informed scientific review. For ashwagandha, despite a significant number of clinical studies, there is little publicly available toxicology data available to demonstrate safety of ashwagandha root and leaf products. We completed a broad range of older legacy but Organization for Economic Co‐operation and Development (OECD) Test Guideline (TG)‐compliant toxicology studies routinely recommended in regulatory safety guidelines specific to the aqueous ashwagandha root and leaf extract, Sensoril. The data summarized in this report support the conclusion that Sensoril is not toxic or genotoxic. No evidence of mutagenicity was observed in the in vitro Ames assay and Sensoril demonstrated negative findings in the in vitro micronucleus assay and in vivo mammalian bone marrow chromosome aberration assay, respectively. Sensoril was examined in rats at dose levels of 1000, 2000, and 4000 mg/kg/day over 90 days and did not exhibit mortality, morbidity, adverse clinical signs or produce test item related changes in weekly body weight, food consumption, ophthalmology, hematological or biochemical parameters. There were no test item related effects on thyroid hormones, sex hormones or microscopic pathology. The data from these studies support a no observed adverse effect level (NOAEL) of 4000 mg/kg/day in rats for an aqueous root and leaf ashwagandha extract.

## Graphical abstract

Sensoril, an aqueous root and leaf extract of ashwagandha showed no evidence of mutagenicity in the in vitro Ames assay, was negative in the in vitro micronucleus, in vivo mammalian bone marrow chromosome aberration assays, and was well tolerated in the rat at up to 4000 mg/kg BW/day when administered orally for a period of 90 days. The data from these studies support a no observed adverse effect level (NOAEL) of 4000 mg/kg BW/day in rats for an aqueous root and leaf ashwagandha extract.

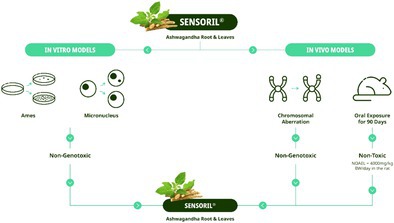

## 1 Introduction

Ashwagandha ( *Withania somnifera* , Dunal Solanaceae), is a member of a family of herbs sometimes referred to as Winter cherry or Indian Ginseng (NIH 2024; Mikulska et al. 2023). Ashwagandha is a low growing evergreen shrub native to India, the Middle East, and parts of Africa (Wiciński et al. 2025). It is cultivated in gardens in the warmer parts of Europe, is a naturalized weed in South Australia and New South Wales, and is grown for its fleshy roots in India (Paul et al. 2021). Traditionally, the roots have been used for over 3000 years in Ayurvedic medicine due to health promoting effects (BfR 2024; Gómez Afonso et al. 2023; Mikulska et al. 2023; Wiciński et al. 2025). Ashwagandha is rich in chemical constituents such as alkaloids, flavonoids, saponins, phenolic acids and withanolides (Shinde et al. 2023; Bashir et al. 2023). Of these constituents, the major bioactive components are the steroidal lactones known as withanolides (Misra et al. 2008; Paul et al. 2021). In addition, each of the plant parts such as leaves, stem, flower, root, seeds, berries and bark may differ in their bioactive constituent content (Paul et al. 2021; Misra et al. 2008).

Herbal parts, including the root, stems, fruits, and leaves, are considered a source of nutrition and have been added to food products (BfR 2024; Krishnamurthy and Sarala 2010; Singirala et al. 2025). Ashwagandha is incorporated into different food products such as ghee, honey, and kombucha (Sharifi‐Rad et al. 2021). Recently, powdered forms of both ashwagandha root and leaves of this plant are added to baked goods, juices, beverages, sweets (candies and snacks), and dairy products at levels ranging from 1% to 10% (Sharifi‐Rad et al. 2021; Singirala et al. 2025; Chaturvedi et al. 2018). In European countries, the leaves, root powder, or dried root pieces have been consumed in teas (BfR 2024; RIVM 2024). Perhaps more commonly, Ashwagandha root and/or leaves have been sold as a supplement in the form of capsules or powder (Sharifi‐Rad et al. 2021; BfR 2024; Singirala et al. 2025).

A 2024 comprehensive review by Basudkar et al. (2024) documents the existing pre‐clinical and clinical data on ashwagandha. Despite a large body of clinical studies, pre‐clinical data is limited. Most of the pre‐clinical studies cited in the Basudkar et al. (2024), review focus on mechanistic aspects of exposure as opposed to the Organization for Economic Co‐operation and Development (OECD) Test Guideline (TG) for the testing of chemicals, which are a library of internationally agreed testing methods used to identify and characterize chemical hazards using toxicology studies (e.g., acute oral study, sub‐acute oral study, a prenatal developmental toxicity study, Ames assays, chromosomal aberration, and micronucleus test). Thus, whilst OECD TG and Good Laboratory Practice (GLP)‐compliant preclinical toxicology studies exist for ashwagandha, the majority of studies examine the ashwagandha root extract (Patel et al. 2016; Wangikar et al. 2024; Kalaivani et al. 2023, 2024; Kalaiselvan et al. 2025) or whole plant extract (Balkrishna et al. 2022) and thus there is a paucity of data investigating the safe use of the ashwagandha root and leaf extracts (or other plant parts) compared to the number of clinical studies investigating the efficacy of Ashwagandha extracts (Mandlik and Namdeo 2021; Kalaivani et al. 2023, 2024; Singirala et al. 2025). This type of public safety data is particularly important considering in real world use, there have been case reports of liver injury linked to consumption of food supplements containing Ashwagandha extracts or commercial herbal products (NIH 2024; RIVM 2024). However, NIH in their review pointed out that although hepatotoxicity concerns could be attributed to withanolides, mislabeling and adulteration with other herbal products cannot be ruled out concluding that Ashwagandha is “considered generally safe and without major adverse effects” (NIH 2024). The work presented herein is important in the continued contribution to properly documenting pre‐clinical safety studies associated with specific plant parts and extraction processes. The present study evaluates the comprehensive toxicity of standardized aqueous extract of root and leaf with the trade name of Sensoril. Completed preclinical studies include acute toxicity, genetic toxicology and an oral 90‐day subchronic toxicology study that followed OECD guidelines for testing of chemicals.

## 2 Materials and Methods

### 2.1 Test Article

The ashwagandha extract used in this study with the trade name Sensoril (Kerry Group, Tralee, Ireland) was a standardized aqueous extract of roots plus leaves of *Withania somnifera* plant (1:1 root and leaf, 100% aqueous extracted, 5:1 Drug Extract (DER) ratio) prepared by a proprietary method. The standardized aqueous extract consists of 50 small molecules, including 26 withanolides. Ten major components (9 withanolides and one flavonoid glycoside) are selected for quantification by ultra‐performance liquid chromatography (UPLC) with photodiode array detection and were within the range of 0.4%–1.0% containing less than 0.5% withaferin A. The test article, Sensoril, used in the Acute toxicity (Batch No. WS1006083), the Ames Test and Mammalian Bone Marrow Chromosome Aberration Test (Batch No. WS0502013), the in vitro Micronucleus test (Batch No. WS202206001‐P‐SNPL), and the 90‐day subchronic study (Batch No. SNL‐0296‐20 from Lot WS202011058 FCL and Batch No. SNL‐0251‐21 from Lot WS202105119‐FCL), was a brown fine free flowing powder that was stored at ambient temperature and protected from light.

### 2.2 Acute Toxicity

The aim of this study was to evaluate acute toxicity of Sensoril after a single oral administration (via intubation canula) in Swiss Albino mice (female) in accordance with OECD TG 423 (OECD 2002). Mice were fasted prior to test compound treatment and were divided into four groups (Group 1, 1A, 1B, 2A, and 2B) with three animals per group. Group 1 served as the control group and was administered distilled water. Group 1A and 1B were administered 300 mg/kg BW Sensoril; Group 1B was administered its dose 24 h after Group 1A received its dose. Group 2A and 2B were treated with 2000 mg/kg BW Sensoril; Group 2B was administered its dose 24 h after Group 2A received its dose. All groups were observed for any signs of toxicity, symptoms, and mortality for the first 30 min and then periodically during the first 24 h. Animals were observed daily thereafter for a total of 14 days. The animals were sacrificed on day 14 and gross morphological features were examined.

### 2.3 Ames Assay

The mutagenic potential of Sensoril was evaluated in vitro using the bacteria reverse mutation assay (Ames Test) in the presence and absence of a metabolic activating system (S9) in accordance with OECD TG 471 (OECD 1997) under Good Laboratory Practices (GLP). The test material was dissolved in DMSO and diluted prior to treatment. Strains employed were as follows: *Salmonella typhimurium* strains TA98, TA100, TA1535, TA 1537, and TA 102, obtained from Xenometrix, San Diego, CA, USA, and *S. typhimurium* strain TA1537, obtained from MOLTOX Inc., NC, USA. Prior to each test, tester strains were incubated for 10 h in nutrient broth at 37°C until 10^9^ cells/mL culture was reached. The S9 liver microsomal fraction was prepared at BSL BIOSERVICE GmbH according to the method of Ong et al. (1980). Male Wistar rats were induced with phenobarbital (80 mg/kg BW) and β‐naphthoflavone (100 mg/kg BW). The S9 mix was stored on ice and subsequently added to the incubation medium and incubated at 37°C during the experiment.

A range finding assay (pre‐incubation method) was conducted with *S. typhimurium* strains TA98 and TA100 at Sensoril concentrations of 3.16, 10, 31.6, 100, 316, 1000, 2500, and 5000 mg/plate. Sensoril concentrations tested in the main experiment using both the plate incorporation method and pre‐incubation method were 31.6, 100, 316, 1000, 2500, and 5000 mg/plate in triplicate. Vehicle control for the assay was DMSO. Positive controls for the assay without metabolic activation were sodium azide (NaN_3_) 10 mg/plate, (Merck, Lot No. K28585088), for TA100 and TA1535; 4‐nitro‐o‐phenylene‐diamine (4‐NOPD), (Fluka, Lot No. 416325), 10 mg/plate for TA98; and 40 mg/plate for TA1537, methyl methane sulfonate (MMS), (Sigma, Lot. No. 123K3690), 1 mg/plate for TA102. Positive controls for the assay with metabolic activation were 2‐aminoanthracene (2‐AA), (Aldrich, Lot No. S11‐804‐114), 2.5 mg/plate for TA98, TA100, TA1535, TA1537 and 10 mg/plate for TA102.

### 2.4 In Vitro Micronucleus Assay

The in vitro micronucleus assay was conducted using the human lymphoblastoid cell line TK6 in accordance with OECD TG 487 (OECD 2023) under GLP conditions. TK6 human lymphoblast cells were obtained from The American Type Culture Collection (ATCC), Manassas, VA, USA. Mycoplasma‐free TK6 cells were maintained in RPMI 1640 medium supplemented with 10% heat‐inactivated horse serum, sodium pyruvate and 1% penicillin–streptomycin culture medium. TK6 cells have a modal chromosome number of 47 and a cell cycle time of 13–15 h, besides being p53 competent, karyotypically stable and DNA repair proficient. Vehicle control for the assay was analytical grade water from a Millipore Elix Essential 10 UV water purification system.

Mammalian post‐mitochondrial fraction (S9) was prepared at Intox Pvt. Ltd., from the liver of male Wistar rats induced with sodium phenobarbital (40 mg/kg in 0.9% NaCl) and β‐naphthoflavone (100 mg/kg in peanut oil) following the method of Westphal et al. (2000). The S9 fraction was stored frozen at −80°C, thawed, and reconstituted in culture medium containing co‐factors prior to use.

Sensoril was tested in the in vitro micronucleus assay using exponentially proliferating TK6 human lymphoblasts in the presence of metabolic activation (S9) for short incubations (3 h) or the absence of metabolic activation for short (3 h) and long incubations (24 h). Positive controls without metabolic activation were mitomycin C (MMC) (160 ng/mL, Sigma Aldrich) and vinblastine (VIN) (3.5 ng/mL, Sigma Aldrich). The positive control with metabolic activation was cyclophosphamide monohydrate (CPA) (6.25 mg/mL, MP Biomedicals).

Range‐finding experiments were conducted to determine a suitable range of Sensoril concentrations for testing in this assay. Multiple concentrations of Sensoril (125–2000 mg/mL) were tested to assess micronuclei formation using the following criteria: (1) highest concentration: inducing approximately 50%–60% cytotoxicity; (2) intermediate concentration: inducing approximately 20%–40% cytotoxicity; and (3) lowest concentration: inducing 0%–20% cytotoxicity. Cell counts were conducted with a hemacytometer, and cytotoxicity was determined using relative population doubling (RPD) per OECD TG 487 guidelines. In the absence of cytotoxicity, the highest concentration was tested. For each test concentration, positive and negative controls were examined in triplicate in the range‐finding and main experiment. The main experiment evaluated Sensoril at 125, 250, and 500 mg/mL.

Following the short (3 h) incubation time Sensoril was removed by centrifugation, the TK6 cells washed and resuspended with complete RPMI 1640 culture medium and transferred to a culture flask for a further incubation period of 18–19 h. Following the long incubation period (24 h) Sensoril was removed by centrifugation, the TK6 cells washed and resuspended with complete RPMI 1640 culture medium and transferred to a culture tube with no recovery period. Cells were harvested using centrifugation, resuspended in complete RPMI culture medium, and prepared for analysis with flow cytometry according to the Litron In Vitro Microflow kit procedure. Micronucleus frequencies were analyzed from ten thousand cells per culture using a BD FACSVerse flow cytometer (BD Sciences, USA).

Statistical Package for the Social Sciences (SPSS Version 23.0) was used for statistical analysis. The percentage of micronuclei (%MN) for each treatment concentration was compared to the vehicle control using one‐way analysis of variance (ANOVA), preceded by Levene's test for homogeneity of variances. Dunnett's multiple comparison test was applied following ANOVA. Additionally, the Kruskal‐Wallis test, followed by the Mann–Whitney test, was used to assess dose–response trends.

### 2.5 Mammalian Bone Marrow Chromosome Aberration

The potential for Sensoril to cause chromosomal aberrations was evaluated using the Mammalian Bone Marrow Chromosome Aberration Test in accordance with OECD TG 475 (OECD 1997) under GLP conditions. Sodium chloride (NaCl) (0.9%) was used as the vehicle and the negative control. The positive control cyclophosphamide monohydrate (CPA) was dissolved in 0.9% NaCl (40 mg/kg, Sigma) and administered as a single intraperitoneal (i.p.) injection. Sensoril was dissolved in 0.9% NaCl 1 h before treatment.

Healthy NMRI mice (6–12 weeks old obtained from Harlan Winkelmann, Borchen, Germany) were weighed prior to treatment to ensure each animal was within 20% of the mean weight of each sex as per OECD TG 475 guidelines (OECD 1997). All animals were examined for clinical signs at various timepoints post‐treatment.

A preliminary toxicity study with Sensoril at 1000 or 2000 mg/kg BW was performed on the same strain under identical conditions as the main experiment using three animals per sex, treated once via i.p. injection at a volume of 10 mL/kg, to identify the maximum tolerated dose, as per OECD TG 475 guidance (OECD 1997).

In the main experiment, five animals per sex were exposed to a single i.p. injection of Sensoril at 200, 500, or 1000 mg/kg BW for an exposure duration of 24 h or a single i.p. injection of Sensoril at 1000 mg/kg BW for an exposure duration of 48 h. Five animals per sex served as the negative control and were dosed with 0.9% NaCl for an exposure duration of 24 and 48 h, respectively. Five male and female mice served as the positive control and received a single 40 mg/kg cyclophosphamide (CPA) dose by i.p. injection and were sacrificed 24 h later.

Four hours prior to sacrifice, animals were injected i.p. with the metaphase arresting agent Colcemid (40 ug). At termination femurs were removed and bone marrow was harvested by cutting off the epiphyses and flushing the marrow out with potassium chloride solution (0.4%). Collected cells were incubated at 37°C for 25 min and fixed with 10 drops of 3:1 methanol: glacial acetic acid as the fixative with continuous mixing. The cell suspension was centrifuged (200 × *g*) for 10 min and the supernatant discarded. The sediment containing cells was resuspended in 4 mL of ice‐cold fixative solution. The fixation procedure was repeated twice. Slides were prepared by dropping the cell suspension onto a clean microscopic slide, flame‐drying and staining with Giemsa (Merck). All slides were independently coded before microscopic examination using a 100X oil immersion objectives. A minimum of 1000 cells per animal were analyzed for mitotic index (percentage of cells in mitosis). At least 100 well spread metaphases per animal were scored for cytogenetic damage (chromosome breaks, fragments, deletions, exchanges and disintegrations) unless a distinct positive result was observed in fewer than 100 metaphases. Gaps and polyploidy were scored but were not included in the calculation of aberration rates.

The mean percentage of aberrant cells observed within each group was compared with historical control data range of negative controls to assess the biological relevance of the findings. In addition, the non‐parametric Mann–Whitney test was performed to evaluate 24‐ and 48‐h post‐treatment aberrant cells (excluding gaps) and mitotic index. Differences from the corresponding negative control were considered to be statistically significant at the 95% confidence level (*p* < 0.05) for each variable examined. Statistical evaluation was used to aid interpretation; however, per OECD TG 475 guideline biological relevance was the primary criterion for interpretation of assay results. Biological relevance and statistical significance were considered together.

### 2.6 Subchronic 90‐Day Oral Toxicity Study

The potential for Sensoril to cause toxicity other than genotoxicity was evaluated in a 90‐day repeat dose oral toxicity study in rats conducted in accordance with OECD TG 408 (OECD 2018) under GLP conditions.

#### 2.6.1 Dose Formulation

Sensoril was formulated as a solution in analytical grade water and prepared freshly on a daily basis at 125, 250, and 500 mg/mL for corresponding dose levels of 1000, 2000, and 4000 mg/kg BW/day. The formulations were stirred at ambient temperature to achieve a homogeneous mixture. Samples of the dosing formulation of Sensoril by Withaferin A content were analysed using a validated analytical method to ensure the concentration of the dosing formulation. For the 90‐day study concentration verification analysis samples were collected from the preparations at day 1, week 6 and day 89 (female rats) or day 90 (male rats) of the study. There were no analytical differences between the formulations collected at each of these study timepoints.

#### 2.6.2 Experimental Design

The study comprised four groups of 10 male and 10 female Wistar IGS outbred (Crl:WI) rats 6–8 weeks of age that were obtained from Charles River Inc. through Hylasco Biotechnology (India) Pvt. Ltd., Telangana, India. One group of rats per sex served as vehicle control and received analytical grade water daily by oral gavage for 90 consecutive days. The test groups of 10 per sex/group were administered Sensoril at dose levels of 1000, 2000, and 4000 mg/kg BW/day by oral gavage daily for 90 days. The doses selected for this study were based on a dose range finding study where five animals per sex were administered identical doses of Sensoril by oral gavage for 14 days and were without abnormal clinical signs or mortality. The 90‐day study also included two recovery groups of 5 rats/sex for the vehicle control and high dose group. Rats in these latter groups were maintained on study for an additional 4 weeks at the end of the 90‐day treatment period.

Prior to the start of the study, animals underwent a seven‐ (males) or eight‐day (females) acclimation period in the study room prior to allocation to the various groups by computer randomization (Provantis Preclinical Software, Version 10.2.3). Rats were housed up to three per sex in solid bottom polypropylene cages with stainless steel grill tops and maintained at 19°C–25°C, 30%–70% humidity under a 12‐h light/dark cycle. Feed and potable filtered (Aquaguard water filter), ultraviolet irradiated drinking water was provided ad libitum, except during the overnight fast where food was withdrawn and water was freely available prior to blood collection and necropsy.

Rats assigned to the 90‐day study underwent detailed clinical observations weekly throughout the treatment and the 4‐week recovery period, with ophthalmology, urinalysis, functional, and neurobehavioral tests performed prior to scheduled necropsy. Body weight was measured on the day prior to initiation of treatment (day 0), weekly thereafter and at necropsy.

Following completion of the treatment, blood samples were collected from all animals through the orbital sinus under light carbon dioxide/oxygen anesthesia for clinical chemistry and hematology assessment. Rats were killed by exsanguination under isoflurane anesthesia followed by macroscopic examination, removal, weighing, and preservation of a range of tissues in neutral buffered formalin. Tissues were processed and sections prepared for microscopic examination using hematoxylin and eosin staining. Histopathological examination was carried out on the preserved tissues and organs of end of treatment (day 91) vehicle control and high dose groups. At necropsy, the stage of the oestrus cycle for all females was determined with vaginal smears. For males, at least one cauda epididymis was reserved for histological examination, and the remaining epididymides were examined for sperm reserves, sperm motility, and sperm morphology.

Rats assigned to the vehicle control and high dose group in the four‐week reversal study also had body weights, food consumption, organ weights, and sperm parameters evaluated, with blood samples taken prior to necropsy for a comprehensive range of hematology and clinical chemistry parameters. After macroscopic internal examination, samples of the testes, epididymides, prostate gland, seminal vesicles, coagulating gland, mammary gland, ovaries, uterus, cervix, vagina, and pituitary gland were taken and fixed in formalin and processed with hematoxylin and eosin staining for histopathological examination.

Clinical chemistry plasma samples were analyzed for individual animals using the Dimension Xpand Plus, Clinical Chemistry System (Siemens Healthcare Diagnostics Inc., Newark, USA) and commercially available diagnostic kits (manufactured by Siemens Healthcare Diagnostics Inc., Camberly, UK or Abbkine Inc., China) for the thyroid hormone assays.

Hematology analysis was performed using ADVIA 2120i Hematology System (Siemens Healthcare Diagnostics Inc., Camberly, UK).

#### 2.6.3 Statistical Analysis

Body weight, food consumption, hematology, clinical chemistry, urinalysis, and organ weight data were subjected to Levene's *F* test for testing the assumption of homogeneity of variance and then subjected to an independent *t*‐test, one‐way analysis of variance (ANOVA), and Dunnett's test. The variance was evaluated at the 5% level of significance.

## 3 Results

### 3.1 Acute Toxicity

During a 14‐day acute toxicity, no mortality was observed in any of the experimental groups at 48 h or at the end of the 14‐day observational period even at the highest dose of 2000 mg/kg BW. There were no morphological, necrotic, or internal hemorrhagic changes in any part of the organs observed in either the treated or control groups. There were no significant differences in individual body weight or organ weights observed in either treated or control groups (data not shown).

### 3.2 Ames Assay

A range‐finding study produced no cytotoxicity at Sensoril concentrations up to 5000 mg/plate (data not shown). In the definitive study, the number of revertant colonies was not increased for any of the tested strains with and without metabolic activation using the plate‐incorporation method (Table 1) or pre‐incubation method (Table 2). An increase in the number of revertant colonies [indicative of mutagenicity] was observed in the positive controls with and without metabolic activation. Accordingly, Sensoril did not cause gene mutations by base pair changes or frameshifts in the genome of the tester strains used and is considered to be non‐mutagenic in this bacterial reverse mutation assay.

**TABLE 1:** Mean incidence of revertant colonies per plate in the Ames assay for Sensoril using the plate‐incorporation method.

Sensoril concentration ug/plate	Bacterial reverse mutation assay									
	Strain									
	TA98		TA100		TA 1535		TA 1537		TA102	
	−S9	+S9	−S9	+S9	−S9	+S9	−S9	+S9	−S9	+S9
	Mean ± SD		Mean ± SD		Mean ± SD		Mean ± SD		Mean ± SD	
0	26 ± 2.9	46 ± 11.4	113 ± 16.3	114 ± 2.9	8 ± 0.6	8 ± 2.0	13 ± 4.4	8 ± 3.1	161 ± 14.6	164 ± 11.5
31.6	27 ± 3.5	36 ± 9.5	120 ± 7.6	101 ± 10.7	9 ± 1.0	8 ± 3.5	14 ± 2.3	16 ± 7.0	175 ± 8.2	191 ± 14.2
100	31 ± 5.7	46 ± 2.5	124 ± 12	104 ± 16.6	8 ± 1.7	11 ± 1.5	15 ± 1.2	15 ± 1.5	182 ± 18.7	206 ± 24.3
316	28 ± 1.0	38 ± 1.5	108 ± 6.2	110 ± 14.2	14 ± 1.7	9 ± 1.5	13 ± 7.0	13 ± 2.1	160 ± 19.2	197 ± 21.1
1000	28 ± 2.1	41 ± 3.8	128 ± 13.7	109 ± 12.2	13 ± 4.9	8 ± 2.9	17 ± 1.5	19 ± 0.6	178 ± 25.2	139 ± 15.6
2500	26 ± 6.1	36 ± 1.2	132 ± 1.2	99 ± 15.1	9 ± 2.3	11 ± 1.5	17 ± 4.5	15 ± 7.1	149 ± 32.1	171 ± 43.3
5000	30 ± 5.8	31 ± 5.0	132 ± 4.0	113 ± 11	13 ± 1.2	11 ± 3.2	12 ± 1.7	18 ± 1.0	207 ± 21.1	206 ± 23.7
Positive Control −S9	842 ± 63.8^a^	—	1083 ± 58.3^b^	—	348 ± 48.2^b^	—	193 ± 34.6^c^	—	1529 ± 337.9^d^	—
Positive Control +S9	—	631 ± 188^e^	—	1230 ± 54^e^	—	71 ± 16.5^e^	—	43 ± 4.2^e^	—	578 ± 72.2^f^

*Note:* Data shown are mean ± SD revertant colonies per plate in triplicate. Positive controls: ^a^4‐nitro‐o‐phenylene‐diamine (4‐NOPD) at 10 mg/plate, ^c^4‐NOPD at 40 mg/plate; ^b^sodium azide (NaN_3_) at 10 mg/plate; ^d^methyl methane sulfonate (MMS) at 1 mg/plate; ^e^2‐aminoanthracene (2‐AA) at 2.5 mg/plate, ^f^2‐AA at 10 mg/plate.

**TABLE 2:** Mean incidence of revertant colonies per plate in the Ames assay for Sensoril using the pre‐incubation method.

Sensoril concentration ug/plate	Bacterial reverse mutation assay									
	Strain									
	TA98		TA100		TA 1535		TA 1537		TA102	
	−S9	+S9	−S9	+S9	−S9	+S9	−S9	+S9	−S9	+S9
	Mean ± SD		Mean ± SD		Mean ± SD		Mean ± SD		Mean ± SD	
0	42 ± 4.7	44 ± 6.4	122 ± 11.6	118 ± 15.4	10 ± 5.1	13 ± 3.8	20 ± 6.4	19 ± 5.2	221 ± 15.8	304 ± 6.4
31.6	29 ± 3.0	38 ± 1.7	134 ± 13.7	118 ± 9.2	18 ± 3.6	11 ± 5.5	19 ± 4.6	20 ± 6.5	232 ± 26.5	295 ± 6.2
100	34 ± 3.0	36 ± 1.7	128 ± 14.4	120 ± 8.7	13 ± 2.1	12 ± 2.1	18 ± 5.1	21 ± 3.8	242 ± 3.6	313 ± 8.5
316	31 ± 7.5	37 ± 2.5	106 ± 8.6	125 ± 13.6	15 ± 3.6	11 ± 3.8	17 ± 2.1	20 ± 7.9	214 ± 4.6	296 ± 38.7
1000	31 ± 1.5	45 ± 6.1	116 ± 15.8	119 ± 18.0	10 ± 0.6	9 ± 1.0	17 ± 3.1	27 ± 8.5	241 ± 13.0	258 ± 24.9
2500	29 ± 13.8	49 ± 9.0	93 ± 2.6	130 ± 3.2	9 ± 1.5	13 ± 3.1	13 ± 1.5	19 ± 2.0	185 ± 22.0	294 ± 41.4
5000	31 ± 6.7	36 ± 6.4	114 ± 6.1	130 ± 9.0	13 ± 2.6	11 ± 2.3	9 ± 1.7	23 ± 1.5	211 ± 13.1	295 ± 31.2
Positive control −S9	1063 ± 146.0^a^	—	950 ± 232.8^b^	—	656 ± 208.6^b^	—	197 ± 15.1^c^	—	2410 ± 158.2^d^	—
Positive control +S9	—	777 ± 19.9^e^	—	1086 ± 93.6^e^	—	68 ± 12.6^e^	—	82 ± 13.1^e^	—	1024 ± 148.2^f^

*Note:* Data shown are mean ± SD revertant colonies per plate in triplicate. Positive controls: ^a^4‐nitro‐o‐phenylene‐diamine (4‐NOPD) at 10 mg/plate, ^c^4‐NOPD at 40 mg/plate; ^b^sodium azide (NaN_3_) at 10 mg/plate; ^d^methyl methane sulfonate (MMS) at 1 mg/plate; ^e^2‐aminoanthracene (2‐AA) at 2.5 mg/plate, ^f^2‐AA at 10 mg/plate.

### 3.3 In Vitro Micronucleus Assay in TK6 Cells

The range‐finding assay was conducted to determine the highest Sensoril concentration that resulted in 50%–60% cytotoxicity. Doses tested were 125–2000 mg/mL. Treatment of TK6 cells for 3 h with metabolic activation produced greater than 60% cytotoxicity at 1000 and 2000 mg/mL (data not shown); consequently, Sensoril concentrations of 125, 250, and 500 mg/mL were selected for the main experiment. Treatment of TK6 cells with Sensoril at 125, 250, and 500 mg/mL for 3 h with and without metabolic activation did not result in a statistically significant increase in the percentage of micronuclei (%MN) relative to the vehicle control group (Table 3). The positive control cyclophosphamide (CPA) and mitomycin C (MMC) statistically significantly increased the number of %MN relative to the vehicle control group following 3 h of incubation with and without metabolic activation, respectively. Incubation of TK6 cells at the same concentrations for 24 h without metabolic activation also did not statistically significantly increase %MN (Table 3). The positive control vinblastine (VIN) statistically significantly increased the number of %MN relative to the vehicle control group following 24 h of incubation without metabolic activation (Table 3). Precipitate formation was not observed for either incubation period. These results indicate that Sensoril is non‐clastogenic and non‐aneugenic under these test conditions.

**TABLE 3:** Summary of Sensoril in vitro micronucleus assay with TK6 human lymphoblasts.

Test item	3‐hour exposure with metabolic activation (S9)		
	Dose group (ug/mL)	% Cytotoxicity	% MN (mean ± SD)
Vehicle control analytical grade water	0	—	1.43 ± 0.37
Positive control CPA	6.25	53.83	5.28 ± 0.07*
Sensoril	125	10.98	1.45 ± 0.16
	250	30.07	1.35 ± 0.18
	500	54.34	1.42 ± 0.04

Test item	3‐h exposure without metabolic activation		
	Dose group (ug/mL)	% Cytotoxicity	% MN (mean ± SD)
Vehicle control analytical grade water	0	—	1.45 ± 0.16
Positive control MMC	0.16	56.31	4.42 ± 0.11*
Sensoril	125	13.67	1.44 ± 0.14
	250	33.70	1.46 ± 0.08
	500	53.84	1.45 ± 0.04

Test item	24‐h exposure without metabolic activation		
	Dose group (ug/mL)	% Cytotoxicity	% MN (mean ± SD)
Vehicle control analytical grade water	0	—	1.53 ± 0.15
Positive control VIN	0.0035	55.31	7.16 ± 0.40*
Sensoril	125	14.13	1.49 ± 0.06
	250	35.75	1.50 ± 0.10
	500	56.52	1.52 ± 0.06

*Note:* Positive controls: CPA – cyclophosphamide monohydrate; MMC – mitomycin C; VIN – vinblastine.

Abbreviations: % MN, percentage micronuclei; SD, standard deviation.

* Significant at *p* < 0.05 one way ANOVA and Dunnett's test.

### 3.4 Mammalian Bone Marrow Chromosome Aberration

The selection of the highest dose group was based on identification of the maximum tolerated dose per OECD TG 475 (OECD 1997). In the preliminary range‐finding study three male and female mice were treated with a single dose of Sensoril via i.p. injection at 2000 mg/kg BW. Two males and one female died within 72 h (data not shown). An additional group of three male and three female mice were treated with single dose of Sensoril via i.p. injection at 1000 mg/kg BW and showed clinical signs of reduced spontaneous activity and closed eyes up to 90 min post‐dose, but without further clinical signs or mortality up to 48 h (Table 4).

**TABLE 4:** Clinical signs following a single intraperitoneal dose of 1000 mg/kg BW Sensoril in range finding study [maximum tolerated dose (MTD)] of chromosomal aberration test.

Observation	Time (post application)			
	5–15 min	60 min	90 min	48 h
Number of animals	3/sex	3/sex	3/sex	3/sex
Reduced spontaneous activity	3M, 3F	3M, 3F	2M, 2F	NA
Closed eyes	0M, 0F	2M, 2F	2M, 2F	NA
Apathy	NA	NA	NA	NA

Abbreviations: F, female; M, male; NA, no significant findings.

In the main experiment, five male and five female mice were treated with Sensoril at 200, 500, or 1000 mg/kg BW. Reduction of spontaneous activity, closed eyes and apathy was observed as clinical signs at both 1000 and 500 mg/kg BW (Tables 5 and 6). No clinical signs were observed in mice treated with Sensoril at 200 mg/kg BW.

**TABLE 5:** Clinical signs following a single intraperitoneal dose of 1000 mg/kg BW Sensoril in main experiment of chromosomal aberration test.

Observation	Time (post application)			
	3–5 min	10–15 min	14 h	38 h
Number of animals	5/sex	5/sex	5/sex	5/sex
Reduced spontaneous activity	12M, 12F	12M, 12F	12M, 12F	7M, 7F
Closed eyes	12M, 12F	5M, 5F	NA	NA
Apathy	12M, 12F	12M, 12F	12M, 12F	7M, 7F

Abbreviations: F, female; M, male; NA, no significant findings.

**TABLE 6:** Clinical signs following a single intraperitoneal dose of 500 mg/kg BW Sensoril in main experiment of chromosomal aberration test.

Observation	Time (post application)			
	3–5 min	10–15 min	14 h	24 h
Number of animals	5/sex	5/sex	5/sex	5/sex
Reduced spontaneous activity	5M, 5F	5M, 5F	5M, 5F	NA
Closed eyes	5M, 5F	5M, 5F	NA	NA
Apathy	5M, 5F	5M, 5F	NA	NA

Abbreviations: F, female; M, male; NA, no significant findings.

In mice treated for 24 h, the mitotic index values remained within historical control limits and did not differ significantly from the negative control and were without statistical significance (Table 7). In the 1000 mg/kg BW Sensoril‐treated mice exposed for 48 h, the mitotic index was statistically significantly increased in both males and females relative to the vehicle control, indicating a cytostatic response (Table 7).

**TABLE 7:** Summary of chromosome aberration assay results for Sensoril.

Dose group	Metaphases	Aberrant cells excluding gaps		Gaps	Polyploid cells	Mitotic index (% ± SD)
		Number	% ± SD			
Negative control 24 h						
Male	500	17	3.4 ± 2.2	11	0	5.26 ± 0.86
Female	500	6	1.3 ± 1.3	9	0	4.84 ± 0.16
Sensoril 200 mg/kg BW 24 h						
Male	500	20	4.0 ± 2.0	12	0	6.08 ± 1.55
Female	500	4	0.8 ± 0.8	4	0	5.62 ± 1.34
Sensoril 500 mg/kg BW 24 h						
Male	500	19	3.8 ± 2.4	6	0	6.32 ± 1.74
Female	500	7	1.4 ± 1.3	5	0	6.76 ± 1.55
Sensoril 1000 mg/kg BW 24 h						
Male	500	22	4.4 ± 0.5	6	0	5.50 ± 1.66
Female	500	15	3.0 ± 2.0	11	0	6.26 ± 2.27
CPA 40 mg/kg BW 24 h						
Male	230	57	24.8 ± 12.3*	10	0	0.68 ± 0.49
Female	365	41	11.2 ± 4.9*	14	0	1.64 ± 0.85
Negative Control 48 h						
Male	500	18	3.8 ± 1.6	3	0	4.54 ± 0.87
Female	500	8	1.6 ± 1.1	6	0	3.84 ± 0.68
Sensoril 1000 mg/kg BW 48 h						
Male	500	19	3.8 ± 1.5	6	0	8.04 ± 1.73*
Female	500	21	2.0 ± 1.2	10	0	6.84 ± 1.40*

Abbreviations: %, percentage; h, hours; SD, standard deviation.

* *p* < 0.05.

Mean values of percentage aberrant cells in the 24‐ and 48‐h male and female negative controls and Sensoril‐treated groups were no different from the concurrent negative control and were within the laboratory historical control range for negative control values (Table 7). The positive control CPA induced the expected increase in chromosomal aberrations (*p* < 0.05) and a decrease in the mitotic index. These results indicate that Sensoril is non‐clastogenic under conditions of the test.

### 3.5 Subchronic 90‐Day Oral Toxicity Study

#### 3.5.1 Mortality, Morbidity and Clinical Signs of Toxicity

During the study there were no incidents of mortality or clinical signs during the 90‐day treatment period. All male and female rats in the 28‐day reversal period survived and were without clinical signs (data not shown).

#### 3.5.2 Body Weight and Food Consumption

**FIGURE 1:**
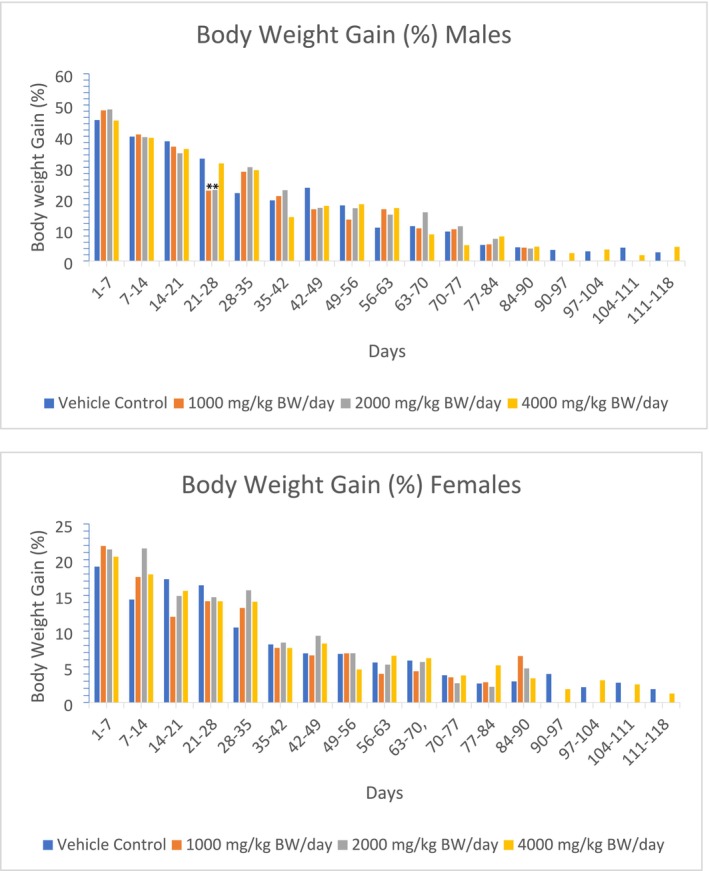
Mean body weight gain (g) in Male and Female Sensoril‐treated rats. *Significant at *p* < 0.05 one‐way ANOVA and Dunnett's test.

From Day 21 to 28, body weight gain was significantly reduced (*p* < 0.05) in the 1000 and 2000 mg/kg BW/day male Sensoril‐treated groups, when compared to the vehicle control (Figure 1). This change was transient, lacked dose‐dependence, and did not occur in any other treatment or recovery period or in female rats. The reduction of body weight gain also did not correlate with a reduction in food consumption from Day 21–28. The reduction in body weight gain is therefore considered incidental. No changes in body weight and body weight gain were observed with Sensoril at 4000 mg/kg BW/day (Figure 1). Food consumption in both genders was comparable in Sensoril‐treated rats and the vehicle control (data not shown).

#### 3.5.3 Ophthalmology and Functional Observation Battery

Daily treatment with Sensoril at up to 4000 mg/kg BW/day for 90 days did not reveal ophthalmological abnormalities at either pre‐test or prior to study termination in male and female rats (data not shown). At the end of the study, no abnormal alterations in sensory reactivity, grip strength, or locomotor activity as part of the neurobehavioral assessments were observed in any Sensoril‐treated rats compared to the vehicle control (data not shown).

#### 3.5.4 Hematological Parameters

Administration of Sensoril did not produce toxicological‐related changes in hematological parameters. However, in the main study groups, a few statistically significant changes were observed, including a lower % eosinophils, neutrophils, MCH, and PT and higher % monocytes, MCHC, and PT in male and/or female Sensoril‐treated animals (Table 8). These changes were sporadic, not dose‐related, and except for PT, platelets, and % monocytes in 4000 mg/kg BW/day treated animals, resolved after recovery and the majority were within the historical control range of the test facility, except for % monocytes in males where all groups, including the vehicle control, were slightly above the historical control data at end of treatment (day 91) and in recovery (day 119) 4000 mg/kg/BW males (data not shown). In addition, hematological parameters did not correlate with adverse histopathology of the spleen or bone marrow, further supporting a lack of an adverse effect.

**TABLE 8:** Effect of 90‐day oral administration of Sensoril on hematology parameters.

Parameter (mean ± SD)	Units	Sensoril dose (mg/kg BW/day)			
		0	1000	2000	4000
Day 91					
*Males*					
HGB	g/dL	15.82 ± 0.54	15.59 ± 0.69	15.70 ± 072	15.31 ± 0.38
PCV	%	47.04 ± 1.91	47.54 ± 1.92	47.43 ± 1.44	46.31 ± 0.93
Total RBC	×10^6^ cells/μl	9.00 ± 0.54	9.31 ± 0.54	9.10 ± 0.50	8.78 ± 0.36
Total WBC	×10^3^ cells/μl	13.00 ± 1.61	12.66 ± 2.38	13.26 ± 1.74	13.02 ± 1.85
NEU	%	26.33 ± 3.50	28.03 ± 3.53	28.93 ± 4.86	27.74 ± 4.31
LYM	%	67.70 ± 3.68	65.38 ± 3.55	64.25 ± 4.90	66.26 ± 5.21
EOS	%	1.42 ± 0.23	1.13 ± 0.40	0.91 ± 0.26*	0.95 ± 0.54*
MONO	%	3.85 ± 0.53	4.46 ± 0.63	5.06 ± 0.40*	4.16 ± 1.13
BASO	%	0.37 ± 0.07	0.36 ± 0.16	0.40 ± 0.08	0.39 ± 0.06
Platelets	×10^3^ cells/μl	975.60 ± 98.70	1004.10 ± 85.20	1020.10 ± 116.90	1011.30 ± 83.90
RETIC	%	2.24 ± 0.44	1.91 ± 0.51	1.95 ± 0.46	2.39 ± 0.50
MCV	fL	52.34 ± 1.43	51.14 ± 2.17	52.19 ± 2.08	52.77 ± 1.88
MCH	Pg	17.61 ± 0.88	17.17 ± 0.94	17.30 ± 0.30	17.45 ± 0.87
MCHC	g/dL	33.62 ± 0.75	33.58 ± 0.80	33.12 ± 0.63	33.07 ± 0.87
PT	s	16.11 ± 1.07	16.07 ± 0.67	15.74 ± 1.47	17.31 ± 0.79*
APTT	s	13.18 ± 0.90	13.46 ± 0.82	13.00 ± 0.92	13.29 ± 0.66
*Females*					
HGB	g/dL	14.91 ± 0.46	15.05 ± 0.51	15.08 ± 0.87	14.72 ± 0.81
PCV	%	43.09 ± 2.11	43.68 ± 1.81	43.88 ± 2.73	43.86 ± 2.03
Total RBC	×10^6^ cells/μl	7.83 ± 0.52	7.93 ± 0.41	8.14 ± 0.52	8.08 ± 0.42
Total WBC	×10^3^ cells/μl	9.15 ± 1.47	9.02 ± 1.67	7.88 ± 1.72	7.47 ± 1.74
NEU	%	26.45 ± 3.04	26.78 ± 5.29	25.47 ± 5.78	23.38 ± 6.30*
LYM	%	68.35 ± 3.07	66.85 ± 5.64	69.07 ± 5.22	71.34 ± 6.29
EOS	%	1.33 ± 0.59	1.55 ± 0.58	1.58 ± 0.67	1.51 ± 0.49
MONO	%	3.02 ± 0.76	3.92 ± 0.24*	3.15 ± 0.91	3.05 ± 0.83
BASO	%	0.32 ± 0.09	0.40 ± 0.15	0.27 ± 0.13	0.27 ± 0.08
PLT	×10^3^ cells/μl	1118.10 ± 118.30	1102.50 ± 110.80	1145.60 ± 111.80	1115.50 ± 157.20
RETIC	%	2.47 ± 0.58	2.29 ± 0.56	2.68 ± 0.45	2.48 ± 0.45
MCV	fL	55.09 ± 1.38	55.11 ± 1.54	53.97 ± 1.27	54.30 ± 1.51
MCH	pg	19.06 ± 0.87	19.02 ± 0.73	18.56 ± 0.70	18.21 ± 0.63*
MCHC	g/dL	34.59 ± 1.02	34.49 ± 0.77	34.35 ± 0.65	35.53 ± 0.65*
PT	s	17.66 ± 0.78	16.71 ± 0.91	15.70 ± 1.72*	17.50 ± 0.60
APTT	s	13.08 ± 0.48	13.54 ± 0.50	12.87 ± 0.43	13.45 ± 0.70
Day 119					
Parameter (mean ± SD)		0	1000	2000	4000
*Males*					
PLT	×10^3^ cells/μl	1053.2 ± 37.40	—	—	989.20 ± 47.50*
PT	s	15.44 ± 0.83	—	—	16.72 ± 0.56*
*Females*					
MONO	%	2.88 ± 2.10	—	—	4.16 ± 0.28*
PT	s	17.22 ± 0.32	—	—	16.24 ± 0.88*

Abbreviations: APTT, activated partial thromboplastin time; BASO, basophils; EOS, eosinophils; HGB, hemoglobin; LYM, lymphocytes; MCH, mean corpuscular hemoglobin; MCHC, mean corpuscular hemogloin concentration; MCV, mean corpuscular volume; MONO, monocytes; NEU, neutrophils; PCV, packed cell volume; PLT, platelets; PT, prothrombin time; RETIC, reticulocytes; Total RBC, total red blood cell count; Total WBC, total white blood cell count.

* Significant at *p* < 0.05 one‐way ANOVA and Dunnett's test.

#### 3.5.5 Clinical Chemistry

Administration of Sensoril did not produce toxicological‐related changes in clinical chemistry parameters. However, a few statistically significant changes were observed in the main study and recovery groups. Specifically, plasma biochemical analysis revealed GGT was significantly increased in the main study Sensoril‐treated males and urea was significantly decreased in the 4000 mg/kg BW/day females, relative to the vehicle controls (Table 9). The increased GGT was not dose‐related and GGT was significantly decreased in the 4000 mg/kg BW/day recovery males when compared to the vehicle control. However, these main study and recovery GGT values were outside of the test facility historical control range, including the value of the vehicle control at both day 91 and 119 (data not shown). Similarly, sodium was significantly increased and potassium was significantly decreased in the 4000 mg/kg BW/day recovery females (Table 9). The reported levels were within the test facility historical control range, except for potassium in 4000 mg/kg/BW/day recovery (day 119) females, which was slightly below the historical control range (data not shown). These findings were not considered to be toxicologically relevant due to inconsistency between genders, sporadic nature of the findings and lack of histopathological correlation to liver injury for GGT or kidney injury for urea, respectively, despite absolute kidney weight being increased in the kidney of 4000 mg/kg/BW/day females.

**TABLE 9:** Effect of 90‐day oral administration of Sensoril on clinical biochemistry parameters.

Sensoril dose (mg/kg BW/day)					
Parameter	Units	0	1000	2000	4000
		*n* = 10	*n* = 10	*n* = 10	*n* = 10
*Males Day 91*					
ALT	U/L	48.80 ± 7.19	49.80 ± 3.93	56.30 ± 17.64	39.70 ± 8.88
AST	U/L	81.60 ± 9.13	80.30 ± 8.07	87.00 ± 14.49	71.40 ± 13.13
ALP	U/L	78.30 ± 25.62	70.90 ± 20.39	76.50 ± 25.77	75.60 ± 22.65
GGT	IU/L	7.00 ± 1.25	13.20 ± 1.23*	11.50 ± 2.32*	10.50 ± 0.85*
TBIL	mg/dL	0.08 ± 0.06	0.07 ± 0.05	0.11 ± 0.10	0.11 ± 0.06
TBA	μmol/L	20.03 ± 15.47	12.19 ± 3.97	19.46 ± 12.26	21.21 ± 16.23
ALB	g/dL	1.07 ± 0.21	1.07 ± 0.09	1.05 ± 0.12	1.06 ± 0.23
GLUC	mg/dL	83.60 ± 13.90	79.90 ± 5.69	77.60 ± 11.11	70.40 ± 12.86
CA	mg/dL	10.45 ± 0.37	10.58 ± 0.28	10.57 ± 0.33	10.58 ± 0.27
PHOS	mg/dL	5.57 ± 0.35	5.99 ± 0.50	5.57 ± 0.34	5.78 ± 0.28
CHOL	mg/dL	65.90 ± 14.07	67.70 ± 19.42	61.20 ± 8.39	61.40 ± 15.15
TP	g/dL	7.23 ± 0.37	7.52 ± 0.38	7.21 ± 0.38	7.28 ± 0.28
Na	mmol/L	140.30 ± 0.82	139.80 ± 0.92	139.70 ± 0.95	140.50 ± 0.53
K	mmol/L	4.26 ± 0.28	4.46 ± 0.27	4.54 ± 0.23	4.25 ± 0.36
TGL	mg/dL	55.80 ± 14.45	62.60 ± 19.48	57.70 ± 18.09	48.80 ± 9.30
CREA	mg/dL	0.45 ± 0.05	0.52 ± 0.11	0.44 ± 0.08	0.42 ± 0.06
GLOB	g/dL	6.23 ± 0.54	6.50 ± 0.48	6.16 ± 0.42	6.22 ± 0.40
A/G	mg/dL	0.18 ± 0.04	0.17 ± 0.02	0.17 ± 0.02	0.17 ± 0.04
BUN	mg/dL	18.90 ± 2.02	19.10 ± 2.33	20.10 ± 3.11	18.20 ± 1.99
UN	mg/dL	40.50 ± 4.34	40.93 ± 4.99	43.07 ± 6.66	39.00 ± 4.26
LDL	mg/dL	12.40 ± 2.84	14.40 ± 3.24	11.90 ± 3.45	11.80 ± 2.70
HDL	mg/dL	60.00 ± 11.79	72.20 ± 13.76	61.30 ± 11.43	59.80 ± 13.16
T3	pmol/mL	5.89 ± 0.46	6.18 ± 0.45	6.29 ± 0.37	6.16 ± 0.38
T4	pmol/mL	255.74 ± 29.31	259.30 ± 36.68	264.67 ± 36.60	275.86 ± 41.63
TSH	mIU/L	3.65 ± 0.38	3.54 ± 0.32	3.70 ± 0.24	3.81 ± 0.22
*Females Day 91*					
Parameter	Units	0	1000	2000	4000
ALT	U/L	42.90 ± 8.01	38.40 ± 11.03	33.70 ± 6.09	41.80 ± 14.46
AST	U/L	83.50 ± 7.15	79.60 ± 10.08	82.60 ± 6.08	78.50 ± 12.28
ALP	U/L	45.80 ± 11.73	47.50 ± 18.79	43.70 ± 22.88	64.40 ± 25.14
GGT	IU/L	12.50 ± 0.85	13.10 ± 1.79	11.90 ± 1.37	11.60 ± 0.97
TBA	μmol/L	35.71 ± 13.90	27.76 ± 15.34	27.12 ± 16.54	19.42 ± 10.27
TBIL	mg/dL	0.07 ± 0.05	0.11 ± 0.07	0.11 ± 0.07	0.11 ± 0.06
ALB	g/dL	1.20 ± 0.17	1.27 ± 0.18	1.23 ± 0.22	1.21 ± 0.28
GLUC	mg/dL	82.50 ± 6.55	83.20 ± 9.21	81.20 ± 7.73	80.40 ± 10.69
CA	mg/dL	10.15 ± 0.41	10.37 ± 0.33	10.17 ± 0.38	10.41 ± 0.21
PHOS	mg/dL	5.10 ± 0.38	4.86 ± 0.63	5.12 ± 0.61	5.16 ± 0.49
CHOL	mg/dL	69.70 ± 9.26	74.70 ± 11.25	65.70 ± 12.94	65.70 ± 12.94
TP	g/dL	7.42 ± 0.17	7.50 ± 0.35	7.44 ± 0.61	7.42 ± 0.33
Na	mmol/L	136.90 ± 1.29	137.10 ± 1.10	137.70 ± 1.77	137.70 ± 1.25
K	mmol/L	3.94 ± 0.28	4.13 ± 0.27	3.96 ± 0.43	4.22 ± 0.21
TGL	mg/dL	42.20 ± 8.07	46.70 ± 11.50	51.70 ± 10.45	53.70 ± 15.88
CREA	mg/dL	0.59 ± 0.09	0.55 ± 0.07	0.50 ± 0.10	0.48 ± 0.07*
GLOB	g/dL	6.22 ± 0.17	6.23 ± 0.31	6.21 ± 0.73	6.21 ± 0.50
A/G	mg/dL	0.20 ± 0.03	0.21 ± 0.03	0.20 ± 0.05	0.20 ± 0.06
BUN	mg/dL	22.00 ± 3.37	18.60 ± 2.01	20.30 ± 1.89	17.90 ± 3.67
UN	mg/dL	47.14 ± 7.21	39.86 ± 4.31	43.50 ± 4.04	38.36 ± 7.85*
LDL	mg/dL	7.40 ± 2.17	8.80 ± 2.20	7.40 ± 2.07	8.10 ± 1.20
HDL	mg/dL	65.40 ± 7.85	69.80 ± 10.14	62.30 ± 5.91	61.90 ± 12.69
T3	pmol/mL	6.92 ± 0.43	7.02 ± 0.50	7.26 ± 0.30	7.58 ± 0.45*
T4	pmol/mL	318.78 ± 11.04	323.48 ± 14.65	322.65 ± 34.55	308.69 ± 24.19
TSH	mIU/L	4.24 ± 0.26	4.10 ± 0.29	4.03 ± 0.20	4.09 ± 0.20
*Males Day 119*					
GGT	IU/L	7.60 ± 0.55	—	—	6.80 ± 0.45*
*Females Day 119*					
Sodium	mmol/L	137.60 ± 1.14	—	—	139.20 ± 0.45*
Potassium	mmol/L	3.76 ± 0.18	—	—	3.46 ± 0.21*

Abbreviations: AG, albumin/globulin ratio (calculated); ALB, albumin; ALP, alkaline phosphatase; ALT, alanine aminotransferase; AST, aspartate aminotransferase; BUN, blood urea nitrogen; CA, calcium; CHOL, cholesterol; CREA, creatinine; GGT, ƴ‐glutamyltransferase; GLOB, globulin (calculated); GLUC, glucose; HDL, high‐density lipoprotein cholesterol; K, potassium, LDL, low‐density lipoprotein cholesterol; NA, sodium, PHOS, phosphorus; T3, triiodothyronine; T4, thyroxine; TBA, total bile acids; TBIL, total bilirubin; TGL, triglycerides; TP, total protein; TSH, thyroid stimulating hormone; UN, urea (calculated).

* Significant at *p* < 0.05 one‐way ANOVA and Dunnett's test.

#### 3.5.6 Urinalysis

Treatment of male and female rats with Sensoril up to 4000 mg/kg BW/day for 90 days did not induce any adverse effects in urine parameters such as microscopic appearance or analytes such as protein, glucose, ketones, occult blood, urobilinogen, nitrite, leucocytes, and bilirubin and were found to be comparable to vehicle control rats at the end of treatment. Specific gravity was non‐dose‐dependently reduced in the treated male rats, and the mean pH was slightly higher in 4000 mg/kg BW/day Sensoril‐treated males (Table 10). These findings were considered incidental due to a lack of findings in urine volume, kidney histopathology, and inconsistency between genders.

**TABLE 10:** Effect of 90‐day oral administration of Sensoril on male rat urinary parameters (day 91).

Parameter	Vehicle control	Sensoril		
Dose (mg/kg BW/day)		1000	2000	4000
Specific gravity (mean ± SD)	1.020 ± 0.003	1.017* ± 0.002	1.017* ± 0.002	1.015* ± 0.000
pH (mean ± SD)	7.90 ± 0.66	8.25 ± 0.79	7.95 ± 0.60	8.55* ± 0.50

* Significant at *p* < 0.05 one‐way ANOVA and Dunnett's test.

#### 3.5.7 Thyroid Function

TSH and T4 were unremarkable in Sensoril‐treated rats at up to 4000 mg/kg BW/day in both male and female rats at the end of treatment and in recovery animals (Table 9). T3 was significantly increased (*p* < 0.05) in the main study 4000 mg/kg BW/day females (7.58 vs. 6.92 pmol/mL control females, Table 9), but this finding was within historical control range of the test facility (data not shown). This finding is considered incidental due to inconsistency between genders and lack of adverse findings in lipid or cholesterol biochemical parameters, thyroid organ weight, or adverse microscopic findings within this organ.

#### 3.5.8 Hormones

The oral administration of Sensoril to male and female rats for 90 days at up to 4000 mg/kg BW/day did not alter FSH, luteinizing hormone or estradiol at the end of treatment or in recovery animals (data not shown). At the end of treatment (day 91), testosterone was significantly (*p* < 0.05) reduced non‐dose‐dependently in all treated males and dose‐dependently in all treated females (Table 11). Notably, testosterone levels in five end of treatment (day 91) and one recovery (day 119) control males were outside of the historical control data (HCD) with two day 91 control males at greater than two standard deviations than the mean value (77.53 and 74.14 ng/mL). An outlier analysis was conducted and no outliers were identified, suggesting a lack of anomalous values (data not shown). In contrast, testosterone levels in high‐dose recovery animals (day 119) were slightly higher, but not significantly different than control animals. In addition, no changes were observed in sperm motility, sperm concentration, sperm morphology, testes histopathology and absolute and relative testes, prostate or seminal vesicles weights for the main study male animals and high dose recovery males, relative to vehicle control males. For females, three end of treatment (day 91) and two recovery (day 119) control female testosterone levels were outside of HCD concentrations. However, there were no changes to vaginal cytology or uterus and ovary weights. Accordingly, these findings were considered incidental.

**TABLE 11:** Effect of 90‐day oral administration of Sensoril on testosterone (day 91).

Males	Testosterone ng/mL (mean ± SD)	Females	Testosterone ng/mL (mean ± SD)
Vehicle control	45.50 ± 21.27	Vehicle control	19.24 ± 4.39
1000 mg/kg BW/day	25.66 ± 8.21*	1000 mg/kg BW/day	15.68 ± 3.03*
2000 mg/kg BW/day	27.45 ± 19.03*	2000 mg/kg BW/day	15.01 ± 3.27*
4000 mg/kg BW/day	23.39 ± 12.75*	4000 mg/kg BW/day	13.20 ± 1.38*
HCD	16.5 to 44.9	HCD	8.8 to 19.7

Abbreviation: HCD, historical control data.

* Significant at *p* < 0.05 one‐way ANOVA and Dunnett's test.

#### 3.5.9 Organ Weights

Absolute heart weight was statistically significantly (*p* < 0.05) decreased in the 4000 mg/kg BW/day males and absolute spleen weight in the 4000 mg/kg BW/day males and absolute kidney and liver weight in the 4000 mg/kg BW/day females were statistically significantly (*p* < 0.05) increased at the end of the main study relative to the vehicle control group (Table 12). Relative organ to body weight was also statistically significantly (*p* < 0.05) increased in the spleen of 4000 mg/kg BW/day males and in the thymus of 4000 mg/kg BW/day females at the end of the treatment phase. Absolute thymus weight was statistically significantly (*p* < 0.05) decreased in the 4000 mg/kg BW/day recovery females. Absolute or relative to body weight alterations were not associated with microscopic findings in these organs and remained within the test facility historical control range, except for relative thymus to body weight in the 4000 mg/kg BW/day females at the end of the treatment (data not shown). This was only seen at the high dose, was without a correlation to increased or decreased body weight and there was a lack of histopathology concern noted, suggests these organ weight changes are incidental. Further, for the liver and kidney, absolute organ weight increases in the absence of microscopic correlation or biochemical evidence of injury (e.g., increased liver transaminases or increased serum creatinine or proteinuria for the kidney) suggest these findings are incidental.

**TABLE 12:** Effect of oral administration of Sensoril on rat organ weight.

Organ	Sex, group	Weight (g) ± SD (vehicle control)
		Treatment group
*End of treatment period day 91*		
Heart, Absolute	Male, 4000 mg/kg BW/day	(1.61 ± 0.14) 1.42 ± 0.13*
Spleen, Absolute	Male, 4000 mg/kg BW/day	(0.93 ± 0.14) 1.13 ± 0.14*
Spleen, Relative	Male, 4000 mg/kg BW/day	(0.19 ± 0.03) 0.23 ± 0.04*
Kidney, Absolute	Female, 4000 mg/kg BW/day	(1.71 ± 0.10) 1.91 ± 0.18*
Liver, Absolute	Female, 4000 mg/kg BW/day	(8.04 ± 0.72) 9.35 ± 1.38*
Thymus, Relative	Female, 4000 mg/kg BW/day	(0.12 ± 0.03) 0.23 ± 0.02*
*End of recovery period day 119*		
Thymus, Absolute	Female, 4000 mg/kg BW/day	(0.34 ± 0.07) 0.45 ± 0.08*

* *p* < 0.05 one‐way ANOVA and Dunnett's test.

#### 3.5.10 Vaginal Cytology

Microscopic examination of vaginal smears at necropsy of main study and recovery females did not show any differences in the stages of the oestrous cycle in Sensoril treated rats relative to the vehicle control (data not shown).

#### 3.5.11 Gross Pathology and Histopathology

In the gross visual inspection of organs of rats, the Sensoril treatment groups did not differ from the vehicle treated animals. Microscopic examination of tissues from the vehicle control and high dose groups did not reveal remarkable histopathological findings attributable to exposure to Sensoril at up to 4000 mg/kg BW/day. A few isolated incidences of findings in the lungs, adrenals and stomach were observed. These were of minimal severity and comparable incidence to the vehicle‐treated group and thus were considered to be unrelated to treatment with Sensoril (Table 13). For the liver, biochemical analysis revealed GGT was non‐dose dependently increased in the main study males and increased absolute liver weight changes were observed in the 4000 mg/kg BW/day females at day 91, respectively, relative to the vehicle control groups. However, microscopic evaluation of the liver was unremarkable, with no changes observed in the liver architecture relative to the vehicle control animals following Sensoril treatment, suggesting the changes for GGT and liver weight are incidental. In addition, to further evaluate the potential relevance of the observed changes in testosterone levels, all Sensoril treatment groups and vehicle control and high dose groups of recovery groups of both genders underwent external peer review by a reproductive histopathology expert for the following tissues: testes, epididymides, prostate gland, seminal vesicles, coagulating glands, mammary glands, pituitary gland in males and ovaries, uterus with cervix, vagina, mammary glands and pituitary gland in female rats. Adverse histopathological findings were not identified (data not shown).

**TABLE 13:** Effect of oral administration of Sensoril on microscopic findings.

Sex	Male rats		Female rats	
Group	Vehicle control	Sensoril	Vehicle control	Sensoril
Dose mg/kg BW/day	0	4000	0	4000
*Incidence*				
Number of animals examined	10	10	10	10
*Findings*				
Lungs				
Hyperplasia, peribronchial, lymphoid	2	0	2	0
Inflammation, interstitium, suppurative, multifocal	1	1	0	1
Adrenals				
Cortical vacuolization, minimal	4	5	0	0
Accessory structure, cortex, unilateral	1	0	0	0
Stomach				
Dilated glands, minimal	0	1	0	0
Colon				
Submucosal lymphoid hyperplasia, minimal	2	0	3	2

## 4 Discussion

The current studies were performed to better understand the toxicological profile of a proprietary root and leaf aqueous extract of ashwagandha with the trade name of Sensoril. No signs of toxicity or mortality were observed in the 14‐day acute toxicity study in mice at doses up to 2000 mg/kg BW. The root and leaf extract in these studies would be slightly toxic based on a median lethal dose (LD50) standard scale regarding toxicity status of test substances (Erhirhie et al. 2018). However, due to the high mortality limitation, the LD50 is now estimated based on alternative procedures designed to reduce animal use and is no longer a required test by regulatory agencies such as the U.S. Food and Drug Administration (US FDA 1988).

The additional work presented and discussed with this particular ashwagandha root and leaf extract further supports a low toxicity profile as evidenced by longer term exposure (90‐day) to ~4 g/kg‐bw/day with no concerning adverse effects. Authoritative bodies typically expect that once an immediate acute toxicity hazard has been dispelled, that genotoxicity next needs to be assessed. We have shown herein a lack of genotoxicity first demonstrated by the non‐mutagenic response in a bacterial test system (Ames assay) with and without S9 metabolic activation. Sensoril also did not induce chromosomal aberrations in the bone marrow of mice or promote micronuclei formation in a human lymphoblastoid cell line (TK6 cells) with and without S9 metabolic activation. Accordingly, Sensoril can be considered to be non‐mutagenic and non‐clastogenic and important contribution considering genotoxicity data should be generated specific to each plant part (Sun et al. 2021) when examining botanical safety. Previous reports on other plant parts (root or whole plant) have further corroborated this finding for ashwagandha aqueous extracts, which demonstrated a lack of genotoxic effect in an Ames assay, chromosomal aberration assay and micronucleus assay for an aqueous ashwagandha root extract (Kalaivani et al. 2024). It is worth noting that a weakness in the in vivo chromosome aberration work reported herein is that the intraperitoneal route of exposure was used which is not the current preferred method (OECD TG 475 2016; U.S. Food and Drug Administration 2007) for an ingredient that is likely to be ingested. However, the intraperitoneal route was an accepted route of exposure for older legacy studies promulgated by OECD guidance (e.g., OECD TG 475 1997) likely due to greater bioavailability and systemic exposure via this route. Other investigators have investigated the suitability of both the intraperitoneal and oral administration routes to form chromosomal aberrations in bone marrow cells when exposed in vivo to a known mutagen, and found both routes induced chromosomal aberrations, cell cycle delay and sister‐chromatid exchange in bone marrow cells (Chatterjee and Shampa 1999). Thus, supporting the intraperitoneal route as suitable for older legacy chromosomal aberration assays. In light of animal welfare considerations and due to the conservative nature of this approach; it would be inappropriate to run this assay under current OECD test guideline expectations when our results demonstrate systemic exposure was clearly achieved and no genotoxicity was observed.

In the subchronic toxicity study, rats received 1000, 2000, or 4000 mg/kg WB/day of an aqueous root and leaf extract of ashwagandha (Sensoril). Minimal changes were observed in body weight gain, a limited number of hematological and clinical chemistry parameters, thyroid hormones, absolute and relative organ weights, and in gross pathology. All these changes were considered incidental and unrelated to treatment. Similar findings were observed in a methanol‐extracted ashwagandha root powder acute and subacute toxicology study in rats, where significant increases in hemoglobin, white blood cell count, and significant decreases in reticulocyte count and prothrombin time were observed (Patel et al. 2016). In contrast, a sub‐chronic aqueous extracted ashwagandha root toxicology rat study showed no effects on mortality, biochemical or hematological parameters, vaginal cytology, organ weights, or histopathology (Kalaivani et al. 2023). Both studies examined ashwagandha at lower doses, at up to 2000 mg/kg BW/day, relative to the current study.

In addition, the oral administration of Sensoril resulted in no remarkable effect on the sex hormones FSH, LH and estradiol in 90‐day treated or recovery animals. The same was not true for testosterone, which was significantly lower (*p* < 0.05) than controls in 90‐day treated animals in both genders, but was slightly higher and not statistically significantly different from controls in recovery animals (day 119). However, it is important to note that testosterone values in treated and control animals (day 91 and day 119) were far outside (higher) of the expected range of that reported for male and female rats (Gibbs 2005; Pujol and Bayard 1979; Falvo et al. 1972), with testosterone levels reported in some females were actually higher than those reported for some males, where normally testosterone levels are expected to be one quarter to one twentieth of the males (Falvo et al. 1972, 1974). These observations, coupled with the relatively small group sizes in the present study compared to numbers of animals needed for robust hormonal assessment (Chapin and Creasy 2012), suggest the testosterone data may not be appropriate for interpretation. In addition, treatment with Sensoril did not alter reproductive tissue organs weights, sperm parameters, vaginal cytology and tissue histopathology, all of which are sensitive indicators of hormonal effects and thus indicates a lack of Sensoril‐mediated effect in reproductive organs. Further, all 90‐day treated Sensoril‐treatment animal and vehicle control and high‐dose recovery animal reproductive tissues underwent external peer‐review by a reproductive histopathology expert who examined the following tissues: testes, epididymides, prostate gland, seminal vesicles, coagulating glands, mammary glands, pituitary gland in males and ovaries, uterus with cervix, vagina, mammary glands and pituitary gland in female rats. Adverse histopathological findings were not identified, consistent with the study findings, further lending support to lack of Sensoril‐mediated effects on reproductive tissues.

To give an assurance of safety in use, a battery of genotoxicity and acute and subchronic toxicology studies were completed under internationally accepted OECD guidelines for the purpose of risk characterization of an ashwagandha root and leaf extract. Under conditions of the studies and toxicological endpoints evaluated, the aqueous root and leaf extract of ashwagandha, with the trade name of Sensoril, was found to be of no genotoxic concern in the genotoxicity studies and was well tolerated up to 4000 mg/kg BW/day when administered orally for a period of 90 days. The No Observed Adverse Effect Level (NOAEL) of Sensoril in this study was found to be 4000 mg/kg BW/day by the oral route in rats.

## Author Contributions

**Mukesh Summan:** conceptualization, investigation, project administration, writing – original draft, writing – review and editing. **Candace L. Doepker:** writing – review and editing. **Gregory P. Brorby:** writing – review and editing.

## Ethics Statement

The studies were approved by Institutional Animal Ethics Committee Pune, Maharashtra, India, and conducted under conditions recommended by the Committee for the Purpose of Control and Supervision of Experiments on Animals (CPCSEA) guidelines. Animal care was performed according to the National Research Council (2011), *Guide for the Care and Use of Laboratory Animals*, The National Academy Press, Washington DC.

## Conflicts of Interest

Funding for these studies was provided by Natreon Inc./Kerry Group. The authors declare the following financial interests/personal relationships. Mukesh Summan: reports a relationship with Kerry Group that includes employment. Candace L. Doepker and Gregory P. Brorby: report a relationship with ToxStrategies that includes employment. Candace L. Doepker and Greg Brorby: report a relationship with Kerry Group that includes consultancy or advisory.

## Data Availability

The data that support the findings of this study are available from the corresponding author upon reasonable request.
